# Nanotechnology in Medicine: From Inception to Market Domination

**DOI:** 10.1155/2012/389485

**Published:** 2012-03-07

**Authors:** Valentina Morigi, Alessandro Tocchio, Carlo Bellavite Pellegrini, Jason H. Sakamoto, Marco Arnone, Ennio Tasciotti

**Affiliations:** ^1^The Methodist Hospital Research Institute (TMHRI), Houston, TX, USA; ^2^European School of Molecular Medicine (SEMM), Milan, Italy; ^3^Fondazione Filarete, Milan, Italy; ^4^Centre for Macroeconomics and Finance Research (CeMaFiR), Milan, Italy; ^5^Catholic University of Milan, Milan, Italy

## Abstract

Born from the marriage of nanotechnology and medicine, nanomedicine is set to bring advantages in the fight against unmet diseases. The field is recognized as a global challenge, and countless worldwide research and business initiatives are in place to obtain a significant market position. However, nanomedicine belongs to those emerging sectors in which business development methods have not been established yet. Open issues include which type of business model best fits these companies and which strategies would lead them to sustained growth. This paper describes the financial and strategic decisions by nanomedicine start-ups to reach the market successfully, obtain a satisfactory market share, and build and maintain a competitive defendable advantage. Walking nanomedicine-product from the hands of the inventor to those of the doctor, we explored the technological transfer process, which connects laboratories or research institutions to the marketplace. The process involves detailed analysis to evaluate the potentials of end-products, and researches to identify market segment, size, structure, and competitors, to ponder a possible market entry and the market share that managers can realistically achieve at different time horizons. Attracting funds is crucial but challenging. However, investors are starting to visualize the potentials of this field, magnetized by the business of “nano.”

## 1. Introduction

Globally defined as the application of nanotechnology to the clinical arena, nanomedicine has its roots in the same basic concepts and principles of nanotechnology; that is, materials with the nanoscale features present unique characteristics, otherwise absent at a macroscopic level [1]. Just as nanotechnology benefits from mathematics and engineering, nanomedicine too has a multidisciplinary nature involving notions and techniques borrowed from biology, chemistry, and physics [2]. As a result of this successful marriage, nanostructure materials display emerging functions that have exceptional benefits when applied to medical devices.

The success of nanotechnology in the healthcare sector is driven by the possibility to work at the same scale of several biological processes, cellular mechanisms, and organic molecules; for this reason, medicine has looked at nanotechnology as the ideal solution for the detection and treatment of many diseases. One of the many applications of nanotechnology to the medical sector is in the field of drug delivery. The advent of protocols and methods for the synthesis, functionalization, and use of nanoparticles and nano-carriers has flooded the scientific and clinic community with new therapeutic approaches from molecular targeting to radiofrequency ablation and from personalized therapies to minimally invasive techniques.

While most members of the investment community are able to grasp the meaning of nanotechnology and can expertly launch and manage a viable product into the market, they are limited in their conceptual understanding of this scientific discipline and the intricate inner workings behind the product's functionality [3]. On the contrary, those involved in the scientific research recognize that nanomedicine is an expansion of nanotechnology but have very little understanding of the business expertise required to develop their technologies into a commercial product [3]. Cooperation is therefore needed between the two factions in order to lead nanomedicine-based inventions to a successful market position.

## 2. Nanomedicine Market

With 76% [4] of the publications and 59% [4] of the patents, drug delivery is the market segment that dominates the nanomedicine sector. In vitro diagnostics represent the second leading field, contributing with 11% [4] of the publications and 14% [4] of the patent filings. According to the European Commission [4] in a global vision, clustering the publications in the three geographical areas USA, Europe, and Asia (Japan, China, South Korea, Taiwan, Singapore, and India), Europe is leading with 36% [4] of the worldwide publications, followed by the USA with 32% [4] and Asia with 18% [4]. Considering all patent applications in the different fields of nanomedicine, USA hold a share of 53% [4], Europe has 25% [4], and Asia 12% [4]. Biopharmaceutical and medical devices companies are well aware of the potential applications of nanotechnology to the healthcare sector, as demonstrated by the increasingly growing partnerships between these enterprises and nanomedicine startups.

**Figure 1:**
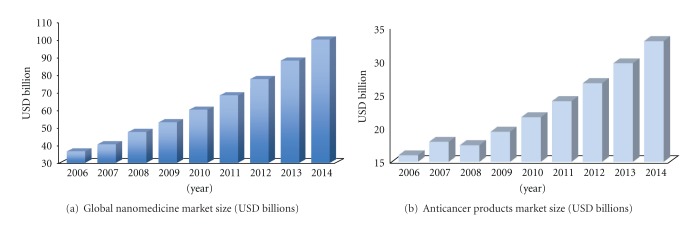
(a) This graph shows the global nanomedicine market size, measured in terms of revenues, such as sales revenues, grants revenues, and milestones. From 2006 to date, a steady growth has occurred, which is expected to continue through 2014, at a CAGR of 13.5% [5]. (b) The graph illustrates the market size for the anticancer applications segment. Except for a slight decrease in 2008, the market has and is predicted to expand by a factor of steady growth [5].

According to a research report from the Business Communications Company (BCC) Research, despite the catastrophic consequences of the 2008-2009 crisis on capital markets, the global nanomedicine sector, which was worth $53 [5] billion in 2009, is projected to grow at a compound annual growth rate (CAGR) of 13.5%, surpassing $100 billion in 2014 (see Figure 1(a)) [5]. One of the largest segments of this market is represented by anticancer products. Valued about $20 billion [5] in 2009, it is expected to reach $33 billion [5] in 2014, growing at a CAGR of 11% [5] (see Figure 1(b)). 

## 3. Financing of Nanomedicine

### 3.1. Common Issues in the Investments on Innovation

The primary output of innovation is obtaining the know-how, which the inventor initially possesses. Unfortunately, the confidentiality of this knowledge can be breached and its use by one company cannot preclude the use of the same by another one. Therefore, investors approaching novel projects are aware of the fact that they will not be able to easily appropriate the total returns of the investment undertaken. As a consequence, there is a lack of attractiveness in financing innovative projects. In fact, from the perspective of economic theory, it is complex to find funding for innovative ideas in a competitive market place [6]. Even in large firms, there is evidence of shortages in resources to spend on the innovative projects that the managers would like to undertake [6]. There are a number of reasons for this phenomenon: low expected returns due to an incapacity to capture the profits from an invention, the exaggerated optimism in undertaking an investment on breakthrough projects, and most notably the uncertainty and risk associated with these projects. Technology-based companies can also consider imitating the inventions developed by competitors. However, Edwin et al. [7], using survey evidence, found that imitating is not costless and could result in expenses equal to 50% [7] to 75% [7] of the cost of the original invention, not eliminating the underinvestment problem. Policymakers are trying to change the funding situation, by facilitating the invention process, rationalizing the interventions through government encouragement of innovative activities, sustaining the intellectual property system, allowing Research and Development tax incentives, and supporting research collaborations. Nonetheless, the path that leads the nanoscale outcome from the laboratory to the marketplace is long and expensive, putting the inventor in a position of disadvantage.

### 3.2. Asymmetric Information, Credibility, and Commitment

The financing and management of innovative products in nanomedicine—like many young and innovative multi-sectoral fields—happens in a context of both financial and product markets failures. These make the financing and management of innovation a particularly complex process, which is also reflected in the corporate governance structure of innovative firms.

Asymmetric information, transaction costs, intangible goods, credibility, and commitment issues, jointly with high and unique risks, make it impossible for traditional financial institutions to be part of the picture, paving the way for angel investors, seed and venture capital investors, or other forms of nontraditional financial institutions.

The asymmetric information issue is partly due to the different information set in the hands of the innovator as opposed to that of the possible provider of funds [8], which gives rise to a “two-sided incentive problem” [9]: the best incentive to reconcile the conflicting behavior of entrepreneur (unobservable efforts) and venture capitalist (monitoring costs) is multistage financing. In an alternative approach, staged financing solves the lack of credibility and of an adequate commitment technology on the part of the entrepreneur.

The credibility and commitment issues arise because the entrepreneur possesses a “unique human capital” [10]: once the Venture Capital has provided financing, the entrepreneur can decide to withdraw and, therefore, hold the VC hostage of his/her decisions. In such conditions, the VC would not provide financing, as the entrepreneur cannot make a credible commitment not to withdraw. The solution in this case is the “staged capital commitment” similar to Hellmann [9] with a different rationale: the unique human capital of the entrepreneurs must be blended with the firms in various sequential stages. This leads to a progressive increase in the expected value of the firm (in terms of a future initial public offering), so that the initial investments become the collateral (the firm itself) for the VC, providing the right incentive to continued financing.

The two approaches also require both the entrepreneur and the VC to participate in the ownership of the firm (as financing happens with shares) and therefore an evolving strategic and managerial relationship between the two parties in an evolutionary view of the firm [11]. Often the VC possesses very good managerial skills, due to its experience in dozens of startups, while the innovating entrepreneur has little or none. Against this backdrop, the staged financing with shares (i.e., joint ownership) also helps addressing the key issue of management decisions: at the beginning of the “relationship,” the entrepreneur has the most detailed technical knowledge and almost complete managerial powers to set up all the technical work that needs to be embodied into the firm. As this knowledge is transferred to the firm, other managerial aspects take priority (e.g., competition, finance, governance) where the VC has better skills. By increasing VC ownership in stages, management powers can be transferred to VC-appointed managers, with specific skill in running an evolving start-up firm and take it adequately to the market, usually with an IPO.

Due to significant concern and disapproval for fundraising in support of innovation, fledgling nanomedicine companies do not have an endless number of financial options. Therefore, in order to establish start-up companies, co-funders generally commit their own money and expertise into it. This is one aspect that represents the internal capital of the startup, as opposed to the external one, which has to be collected from other sources. At this stage start-up companies turn towards government and foundations' grants (i.e., the National Institutes of Health, and the National Science Foundation programs), in order to finance the research and development of their innovative products. These funds are also intended to protect the intellectual property of these novel discoveries and to attract professional investors.

In order to expand and sustain their business, nanomedicine startups usually begins by turning to angel investors—private financiers who provide seed funding—then to venture capitalists (VCs). The interaction and support of these professional investors is essential to assess whether a market entry is possible and to decide which market share managers can realistically achieve at different time horizons. In fact VCs enter at a specific moment of the life of the company when it is still in an early stage, but has already strongly proved its value and perspective. According to Paul A. Gompers and Yuhai Xuan, the general role of VCs is to alleviate asymmetric information between private venture capital-backed targets and the public acquirers, building a bridge between the two parts [12]. These funds plan investment decisions in order to decrease possible agency costs that afflict young entrepreneurial companies. Venture capitalists usually add value to companies in which they invest beyond pure financing, providing managerial expertise, industrial experience, contacts and—not least—momentum [12]. There is strong evidence of VCs involvement in the management of the financed nanotechnology companies as they often have higher costs and longer development times compared to an equivalent information technology business. Furthermore, Baker and Gompers [13] asserted that venture capital-backed firms have better boards of directors compared to those not financed by VCs. This evidence confirms the crucial role played by VCs in the economic success of nanomedicine-based products.

Corporate finance literature has devoted a meaningful stream of research to the relevance of board composition as a useful tool against different typologies of asymmetric information and agency costs. The literature has clearly underlined the existence of a connection between firms' performances and board composition. However, notwithstanding these important results, there is not a universally accepted evidence about the optimal board composition that allows the minimization of the above-mentioned agency costs. In the VC literature evidence, a board composed by internal, external, and instrumental [14] should achieve the result of the minimization of agency costs that is a propaedeutic step for a feasible way out for VC investors.

### 3.3. Landscape

**Figure 2:**
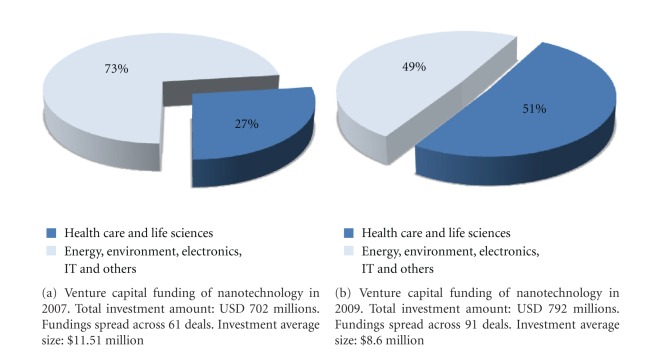
Venture capital investors. Captivated by the great potential of future development, in only two years VCs have shifted their focus on the “science of the tiny things”, nearly doubling investments in this sector.

In 2007 investment in nanotechnology by VCs was US $702 million [15], involving 61 deals. 27% [15] went to healthcare and life science, 31% [15] to energy and environment, and 42% [15] to electronics and IT. Two years later, nanotechnology market captured US $792 million from VCs [15]. Of these, the largest share (51%) [15] went to healthcare and life sciences, followed by energy and environment and electronics and IT, with 23% and 17%, respectively [15]. Doubling the funds invested in the healthcare segment in just two years, the VC industry has demonstrated a clear interest in investment opportunities in the nanomedicine field (see Figures 2(a) and 2(b)). 

Although venture capital investors want to continue to be involved in the science and technology of the small scale, they are extremely cautious about large investments in nanotechnology and nanomedicine, as positive returns on investments are expected only in the long term, especially for nanomedicine [3]. VCs and private investors are still burned by the subprime crisis of 2008 [16], which took a serious toll on their assets, causing catastrophic losses to the whole financial community and restricted access to funds. However, the decline of fundraising might also be a result of ordinary funding cycles, with several VCs having already raised enough resources for the short term [17]. Experts see the Wall Street's crisis of 2008, as a possible regime change [16], rather than a temporary market malfunctioning. After four decades of fairly straightforward access to relatively inexpensive capital, capital markets are currently undergoing major changes [16]. According to the National Science Foundation, innovation is an essential source of competitiveness for economy [18] and represents an excellent opportunity to sustain the economic recovery after the 2008 crisis. As usually happen after a crisis, investors become risk adverse, adopting more rigid risk-cover policies, but there is evidence that the nanobusiness seems to be too attractive not to invest in.

## 4. Business Strategies

The main business area characterizing a nanomedicine company, as well as pharmaceutical and biotechnology industries, is the research and development (R&D). Choosing the R&D strategy, managers evaluate two possible options. The first is based on the idea to perform the entire process inside the company, composing a highly experienced team of scientists. The second option is based on universities or research institutes and is founded on the reliance on leading academic laboratories created over time by “scientific stars.” This second possibility will certainly reduce company costs as these academics frequently cofound the companies based on their discoveries and become part of the scientific boards. We have gathered strong evidence of this second option for the R&D strategy in the companies we analyzed. The commercialization of the research-based product might represent another business area of the nanomedicine company. However, the typical option considered and adopted by managers is to license out the manufacturing and commercialization of the nanomedicine-based product to larger companies. If this is the case, the business model pursued will not include commercialization, and the company will be technology and research based.

The commercialization of the nanomedicine products/technologies is currently driven by startups and small-medium enterprises (SMEs) [4], and it is performed through three types of business models.

(1) *The development of a nanotechnology platform that can be used to add value to second-party products*: this business model seems to be particularly attractive for drug delivery companies, which typically license their particular technologies out to pharmaceutical industries. Otherwise the drug delivery system is tailored and applied to a specific drug complying the particular instructions of the larger company [4].

(2) *The development and manufacturing of high-value materials for the medical device and pharmaceutical industry*: several startups and SMEs merely provide nanomaterials for the manufacture of medical devices or nanotechnology-enhanced drugs [4].

(3) *The development of nanotechnology improved medical devices or pharmaceuticals*: companies adopting this business model intend to develop a proprietary product pipeline as well as trying to bring to the market place new or standard drugs delivered with a drug delivery system or else to develop, for example, a new diagnostic platform based on nanotechnologies [4].

## 5. Regulatory Risk

The US Food and Drug Administration's long approval procedure and regulations make nanomedicine products different from those of other industries using nanotechnologies with no limitations due to regulatory bodies. As a consequence, the expenditure to bring a nanomedical product to the marketplace is so huge that pharmaceutical and biotechnology industries have no alternative but focus on the blockbusters that can please the stockholders [3]. Nanoparticles are not inevitably hazardous, but they have unique properties that question their safety. It is reasonable to presume that nanomaterials are “new for safety evaluations purposes” [3], and therefore they merit careful regulatory oversight by FDA both before and after entering the marketplace. In this arena, federal agencies like the FDA and the US Patent and Trade Mark Office (PTO), impose a sort of order, for the protection of the population safety, while encouraging the development of these products.

The advent of nanomedicine, beside causing changes in the biopharmaceutical industries' business model and value chain, brought two crucial regulatory issues: difficulties in product classification and a lack of scientific expertise on the part of the FDA [19]. 

On the basis of the product's principal method of action, the FDA classifies nanoproducts as drugs, devices, or combination thereof. For regulatory purposes, the FDA applies the same requirements to each part of the combination product and verifies whether the manufacturer gave the correct definition to the product. The definition becomes extremely ambiguous novel for nano-based drug delivery devices as they can be considered either devices (carriers) or drugs (effectors) [19, 20]. The FDA will face exceptional challenges in efficiently regulating such products. In order to successfully do so, a strong scientific knowledge of the field is essential together with a better understanding of the potential risk associated to the exposure of patients to nanomedical products [19].

## 6. Best Practices in the Clinic

Bringing new products to the market has always represented a great challenge, especially when it comes to highly innovative products with high risk/high return. Despite the numerous entry barriers of the nanomedicine market, there are some noteworthy examples of nano-based FDA-approved products that successfully reached the market, impacting medicine and anticipating a change in the healthcare arena.

Within the anticancer products segment, Doxil and Abraxane are two main examples of success in the clinic. Sequus Pharmaceuticals was the first company to sell doxil, the liposomal formulation of Doxorubicin, a powerful but toxic chemotherapeutic, initially approved for treatment of Kaposi's sarcoma in the USA in 1995 [21]. Sequus was then acquired in 1998 by ALZA Pharmaceutical for US $580 millions [22], which subsequently merged with Johnson and Johnson in 2001 in a US $12.3 billion deal [22]. The other approved nanotherapeutic agent, Abraxane, instead, was originally sold by Abraxis Biosciences, which was acquired in June 2010 by Celgene Corporation for US $2.9 billions [23]. Granted by the orphan drug designation in January 2005 by the FDA, this product consists of albumin nanoparticles containing paclitaxel, and is indicated for the treatment of breast cancer [21]. Conventional chemotherapies consist of injections of cytotoxic drug intravenously, which indiscriminately kill both healthy and tumor cells. The clinic success of Doxil and Abraxane was driven by their ability to concentrate preferentially in tumors, because of the gaps (otherwise called endothelial fenestrations) characterizing the blood vessels that supply the cancerous mass. Nanoparticles of the right size can penetrate these “gates” and passively diffuse into the tumors [24]. Thanks to this generation of chemotherapies, patients are now benefiting from new treatment strategies for delivering drugs through nanotechnology carriers with lower systemic toxicity and improved therapeutic efficacy [21].

The economic success of these nanomedical products is driven by an urgent demand of new anticancer therapies able to better fight this highly aggressive and increasingly frequent disease. In fact, the FDA problematic regulatory process, the unsteady funding situation, and the expensive and lengthy R&D process did not thwart the development and success of Doxil and Abraxane.

Despite being the most profitable, anticancer delivery systems are not the only clinically approved nanomedical products. In fact, advances in nanomedicine are bringing breakthroughs in other problematic areas of medicine. Following are some examples of successful nano-enabled biomedical products currently on the market.

The first successful application of nanoparticles in the clinic was Omniscan, the leading injectable paramagnetic resonance product of Amersham. This contrast agent was approved for magnetic resonance imaging (MRI), launched in 1993, and utilized ever since both in neurology, to detect strokes and brain tumors, as well as in cardiology. This contrast agent—originally developed by Salutar—has prolonged half-life in patients with renal insufficiency. After the conduction of preclinical testing, Salutar was acquired by Nycomed, which in turn purchased Amersham International, in 1997. Currently, Amersham and its rights on Omniscan are propriety of General Electric Healthcare. The deal was closed in 2003 for US $9.5 billion on an all-stock transaction. According to Yan et al. [25] and as confirmed by Spiess [26], there are 12 different MRI contrast agents currently on the market [27]. Magnevist was marketed by Bayer Schering Pharma as their first intravenous contrast agent employed in the clinic. In 2004, the company demonstrated that the product safely and effectively eases the visualization of cranial and vertebral anatomy among cancers and wounds, and since then it is diffused worldwide with that specification of use [28]. Another competitor is OptiMARK, a gadolinium-based contrast agent (the only FDA-approved for administration by power injection) for MRI of brain, liver, and spine [29] produced by Mallinckrodt; it allows the visualization of lesions with atypical vascularity. Finally, MultiHance is the first extracellular fluid contrast agent to pose interaction with plasma proteins. Bracco Group produces this contrast agent—an Italian company specialized in diagnostic imaging, drugs and devices—and is utilized in diagnostic MRI of the liver and central nervous system (CNS). It was launched in Europe in 1998 and received the FDA approval for market the product in the United States in 2004 [30].

Returning to the segment of the pharmaceutical applications of nanomedicine, it is important to remember the two FDA-approved nanoparticles-based drugs applied for the treatment of severe fungal infections: AmBisome (liposome for injection), sold by Gilead Sciences and Fujisawa Healthcare and Abelcet (lipid complex), marketed by Elan Corporation. Liposomal formulation of amphotericin B (AmBisome, in its trade name) was originally one of the income-making drugs of NeXstar Pharmaceuticals. The company, along with its products portfolio, was then acquired by Gilead in March 1999. For what concerns Abelcet (the conventional amphotericin B), its North America rights were acquired by Enzon Pharmaceuticals in 2002, in an operational and profitable deal of $360 million (including facilities and operating assets related to the development, production, and sale of the drug). The drug was employed in the treatment of patients with aggressive fungal infection associated to cancer, organs' transplantation, and other postsurgical complications [31]. We wanted to emphasize these two specific products also because they have been subject of a “pharmacoeconomic study.” As a result of the analysis, that involved the two drugs in the empirical treatment of persistently febrile neutropenic patients with presumed fungal infection, AmBisome was found to be more cost-effective compared to Abelcet [32].

RenaZorb sold by Spectrum Pharmaceuticals represents another case of a nano-enabled product, which fruitfully reached the marketplace for the treatment of hyperphosphatemia in end-stage renal disease (ESRD) and potentially chronic kidney disease (CKD). RenaZorb is a lanthanum-based phosphate-binding agent currently in clinical trial, utilizing Spectrum's proprietary nanoparticle technology [33]. The economic and clinical success of this nanoparticle is mainly driven by the clinical scenario. According to the National Kidney Foundation, only in the US are estimated to be more than 20 million people with CKD with numbers expected to double over the next decade. These patients live on kidney dialysis and are potential candidates for phosphate binder therapy [34].

In the light of all this overview of the best practices in the clinic, anticancer remains the biggest share of the nanomedicine market, besides for number of publications and patents, also for number of commercialized products. Increasing acceptance with the general public of the employment of nanotechnologies in the clinic, along with popular widespread sensitivity for the aggressiveness of cancer, can be considered strong drivers for the commercial success of this segment. Furthermore, the first tangible considerable returns due to commercial triumphs represent an undoubted source of attraction for investors. On their part, financiers must realize the importance of providing the substantive funds, necessary to gain the solid results and successful drugs as well as devices and therapies the market requires. The effective investments on Doxil and Abraxane, as well as on the other mentioned successful products, are prime examples of this practice.

## 7. Conclusions and Future Promises

Despite the issues nanomedicine still has to face, investments in this market are predicted to increase. New applications of nanomedicine have been demonstrated, and the resulting expansion of the potential market makes the risk more appealing. Ferocious financial collapse elevated sunk costs of the essential R&D process, tricky access to funds, uncertainty of expected returns, and the extremely meticulous, and lengthy FDA regulatory process has not deterred the investors' community. On the other hand, the promises of great future potential developments in the different market segments and high returns connected to the high risk of the innovation investments make this market still considerably attractive. Compared to the 2007 benchmark, VCs in 2009 decided to double their investments in this sector, at the expenses of the information technology market. The fact that nanomedicine dominates the VC funding in the healthcare market is surely a good predictor of the bright future landscape of expansion of this promising area of research.

Moreover, good returns could even be the result of more accurate assessments of the investments' risks. A pharmacoeconomic analysis would allow the efficient allocation of the monetary resources and the maximization of the highest health return at the lowest costs. A cost-effectiveness analysis (CEA) is structured with a comparison of the costs and effects of two or more treatments, which are under examination. Whereas in the very early stage of the drug development cycle the high failure rate for novel drug molecules is largely due to a not adequate therapeutic index, in the clinical development phase, this rate originates from economic reasons. Therefore, the development of unsuccessful drugs has to be abandoned very fast, in order to save resources for more promising compounds. This saving is obtained through an accurate economic evaluation performed in the early stages of the development process. The benchmark is represented by life-years saved by the investigated nanotherapeutic; if a nano-enabled therapy does not save sufficient life-years to break-even, it should not be developed further [35].

The major limit to the success of this kind of analysis is given by the scarcity of clinical data concerning nanomedicine. The best solution to this issue is collaboration. According to Bosetti and Vereeck [35], economists and investors specialized in health market should work closely with healthcare providers, researchers, patients associations, doctors, and technologists of all kinds, to create a shared platform able to facilitate communication between parties with the ultimate aim to reduce the high risks associated to investments in nanomedicine. As a result, also patients will benefit from these investments, in terms of innovative techniques, therapies, devices, and drugs designed to extend and improve their lives.
